# Non-small Cell Lung Cancer in Women: Experience From the National Institute of Oncology, Rabat, Morocco

**DOI:** 10.7759/cureus.99593

**Published:** 2025-12-19

**Authors:** Boutaina Cherkaoui, Ibrahim El Ghissassi, Intissar Belrhali, Samia El Hilali, Saber Boutayeb, Hind Mrabti, Hassan Errihani

**Affiliations:** 1 Oncology, National Institute of Oncology, Faculty of Medicine and Pharmacy, Mohammed V University, Rabat, MAR; 2 Laboratory of Biostatistics, Clinical Research and Epidemiology, Department of Public Health, Faculty of Medicine and Pharmacy, Mohammed V University, Rabat, MAR; 3 Laboratory of Community Health, Department of Public Health, Faculty of Medicine and Pharmacy, Mohammed V University, Rabat, MAR

**Keywords:** adenocarcinoma, egfr mutations, egfr tki, metastases, non-smokers, nsclc, platinum chemotherapy, survival, women

## Abstract

Introduction and aim

Lung cancer is the most common cancer and the leading cause of cancer deaths worldwide; its incidence in the female population is increasing. The objective of this study was to describe the epidemiological, clinical, paraclinical, therapeutic and evolutionary profile of female patients with non-small cell lung cancer (NSCLC) and to research the predictive factors of better survival.

Methods

A retrospective cohort study was carried out among women with NSCLC, diagnosed between January 2019 and December 2023, at the National Institute of Oncology, Rabat, Morocco. The diagnosis was proven by pathological analysis of biopsy. Statistical analysis was carried out using Jamovi software version 2.5.3.

Results

We assessed 146 women (18.3%) among 805 cases with NSCLC; the average age was 63.5 ± 12.3 years. The percentage of non-smokers, passive smokers and active smokers was 87.7%, 7.5% and 4.8%, respectively. Cough (56.2%), chest pain (21.2%) and dyspnea (19.9%) were the major clinical signs. The common histological types were adenocarcinoma (90.4%), squamous cell carcinoma (6.8%) and other types (2.8%). The primary tumor site was in the right lung in 78 patients (53.4%), on the left in 65 cases (44.5%) and in three cases (2.1%) bilateral. Most patients, 87% (127 cases), were diagnosed at stage IV according to the TNM classification 8th edition.

The epithelial growth factor receptor (EGFR) mutation test performed in 79 female patients (54% of cases) was positive in 28 patients (35.4%). Mutations were predominant on exon 19 (24 patients or 86%), followed by the L858 mutation on exon 21 (four patients or 14%). The anaplastic lymphoma kinase mutation, which was sought in 65 patients (44% of cases), was positive in six patients (9.2%). The search for the reactive oxygen species (ROS1) mutation was performed in seven patients (4.7%) and was positive in one case (14.2%). Programmed death-ligand 1 (PD-L1) expression was affected in 67 patients (45.8%); it was <50% in 57 patients representing 85% of cases and ≥50% was noted in 10 patients (15%).106 stage IV female patients (72.6%) had received first-line palliative systemic treatment, including 89 cases (84%) who had received platinum-based chemotherapy (including 11 EGFR mutated) and 17 EGFR mutated (16%) received anti-EGFR treatment.

In our series, the median overall survival (OS) was 17 months (12.23) and the median progression-free survival (PFS) was 11 months (6.12). Median OS of EGFR-mutated female patients treated in first-line with EGFR TKIs (tyrosine kinase inhibitors) was similar compared with that of EGFR-mutated patients treated with platinum-based chemotherapy (25 months), and median PFS in these patients was slightly longer but statistically non-significant compared with that of patients treated with platinum-based chemotherapy (9.5 months vs. 6.5 months, p=0.22).

Conclusion

The population of women with NSCLC studied was characterized by non-smoking, adenocarcinoma histological type dominance, and a high percentage of EGFR mutations. The median PFS of patients treated with EGFR TKIs was slightly longer than that of patients who received platinum-based chemotherapy. These results encourage us to perform EGFR mutation testing in all patients diagnosed with adenocarcinoma so that they can receive targeted therapy with EGFR TKIs.

## Introduction

Lung cancer is the most common cancer worldwide [1]. In developed countries, mortality rates among women have increased substantially in recent years, in contrast to the stabilization or decline observed in men [2,3]. In Africa, the incidence of lung cancer is rising, particularly in North Africa [1,4,5]. Morocco has a notably high incidence rate (31.9/100,000) and the highest mortality rates in Africa and the Middle East and North Africa (MENA) region, with 7,353 new cases and 6,551 deaths reported in 2020 [6,7].

Lung cancers are histologically classified into non-small cell lung cancer (NSCLC) and small cell lung cancer (SCLC), with approximately 85% of cases corresponding to NSCLC [8]. The primary risk factor for lung cancer is tobacco smoking, with a well-established dose-response relationship [9]. Among women, although smoking remains the principal cause of lung cancer in most developed countries, epidemiological studies have highlighted the involvement of additional epidemiologic, hormonal, genetic, and molecular factors that may act as independent risk factors or interact with the effects of tobacco exposure [10].

The hypothesis that women may be more susceptible to the carcinogenic effects of tobacco remains controversial [11]. Furthermore, the relative importance and contribution of environmental and lifestyle-related factors may vary depending on geographic region and socioeconomic conditions. Lung cancer in women therefore represents a distinct and specific entity that requires further investigation to better characterize its epidemiological, clinical, molecular, and therapeutic features.

In the case of NSCLC, surgery is the treatment of choice for localized stages, whereas chemoradiotherapy remains the standard approach for locally advanced disease. Systemic treatments are used in metastatic stages. Therapeutic strategies for advanced disease have progressively shifted from empirical chemotherapy to a personalized approach based on tumor histology and molecular biomarkers. Despite these advances, the prognosis remains poor, with overall five-year survival rates ranging from 6% to 14% in men and from 7% to 18% in women [12].

In Morocco, very few studies have focused specifically on lung cancer in women [13]. The available studies were conducted in the regions of Rabat, Casablanca, Fes, and Marrakech. In this study, we present a retrospective analysis of female patients diagnosed with non-small cell lung cancer and treated at the National Institute of Oncology in Rabat, Morocco. The objective of this work was to assess the epidemiological, diagnostic, molecular, therapeutic, and prognostic characteristics of NSCLC in women.

## Materials and methods

A retrospective cohort study was conducted at the National Institute of Oncology in Rabat, Morocco, and included all women diagnosed with non-small cell lung cancer (NSCLC) between January 2019 and December 2023. The study population comprised exclusively female patients who had received a confirmed diagnosis of NSCLC based on histopathological examination of biopsy specimens analyzed in the institution’s pathology department. To ensure the homogeneity and reliability of the dataset, several exclusion criteria were applied: male patients, women diagnosed with small cell lung cancer, non-Moroccan patients, and cases with incomplete or non-exploitable medical records were not considered eligible.

Given its comprehensive design, the study included the entire cohort of eligible patients identified over the five-year period, without sampling or case selection. Data were retrieved from the institution’s electronic medical record (EMR) system using a standardized extraction form specifically developed for this study. This instrument enabled the systematic and exhaustive collection of epidemiological, clinical, paraclinical, therapeutic, and follow-up variables, thereby ensuring consistency across records and supporting a robust and reproducible analysis of the patient cohort.

For the statistical analysis, the descriptive analysis was conducted, qualitative variables were expressed as percentage counts and quantitative variables were presented as mean standard deviation because the distribution was Gaussian. The survival analysis was carried out. Progression-free survival (PFS) was defined as the time from the start of treatment to the first documented disease progression or death from any cause. For PFS, Kaplan-Meier curves were constructed and compared by using a log-rank test. Data were entered and analyzed using the statistical software Jamovi version 2.5.3. A p-value <0.05 was considered statistically significant.

Our study was conducted in accordance with the ethical principles outlined in the Declaration of Helsinki and the national regulations governing research involving human subjects. Prior to data collection, the study protocol was reviewed and approved by the Ethics Committee of the National Institute of Oncology in Rabat (approval number: 56/2024). All clinical data were collected retrospectively and handled with strict confidentiality. Informed consent was obtained from all participating patients or, when required, from their legal representatives. No identifiable personal information was used, ensuring complete anonymity of the participants.

## Results

We assessed 146 women (18.3 %) among 805 cases (men and women) of non-small cell lung cancer, and the average age was 63.5 ± 12.3years. The percentage of non-smokers, passive smokers and active smokers was 87.7%, 7.5% and 4.8% respectively (Table 1). More than half of the patients (61%) had a World Health Organization (WHO) performance status of one. Cough (56.2%), chest pain (21.2%) and dyspnea (19.9%) were the main symptoms.

**Table 1:** Demographic, clinical and histological characteristics of patients PS: Performance Status

	Cases (N=146)	Percentage %
Age group		
20-39	7	4.8
40-59	41	28.1
60-79	86	58.9
+80	12	8.2
Smoking status		
Never smokers	128	87.7
Passive smokers	11	7.5
Smokers	7	4.8
Symptoms		
Cough	82	56.2
Chest pain	31	21.2
Dyspnea	29	19.8
Others	4	2.8
Performance Status		
PS0	8	5.5
PS1	89	61
PS2	29	19.9
PS3	8	5.5
PS4	12	8.1
Stage		
Stage IV	127	87
Stage III	17	11.6
Stage II	2	1.4
Stage I	0	0
Non small cell lung cancer type		
Adenocarcinoma	132	90.4
Squamous cell carcinoma	10	6.8
Others	4	2.8

Histological diagnosis was established by CT-guided transparietal biopsy (41%), bronchoscopy (39%) or biopsy of the metastatic site (20%). The common histological types were adenocarcinoma (90.4%), squamous cell carcinoma (6.8%) and other types (2.8%) (Table 1). The site of the primary tumor was in the right lung in 78 patients (53.4%), on the left in 65 cases (44.5 %) and three cases (2.1%) bilaterally.

Most of the patients, 87% (127 cases), were diagnosed at stage IV according to the TNM classification 8th edition, stage III was present in 11.6% of patients (17 cases), two patients (1.4%) were diagnosed at stage II and no patient was diagnosed at stage I (Table 1); 62% of our patients had more than one metastatic site. The common metastatic sites were pleural (30%), bone (26%), lung (22%), brain (21%), liver (16%), lymph nodes (11%) and adrenal (6%).

The epithelial growth factor receptor (EGFR) mutation test was performed in 79 patients (54% of cases), which was positive in 28 patients (35.4%) and negative in 51 patients (64.6%). The mutations were predominant on exon 19 (24 patients or 86 %), followed by the L858 mutation on exon 21 (four patients or 14%) (Table 2). The anaplastic lymphoma kinase (ALK) mutation, which was sought in 65 patients (or 44% of cases), was positive in six patients (9.2%). The search for the reactive oxygen species (ROS1) mutation was performed in seven patients (4.7%) and was positive in one case (14.2%) (Table 2). For programmed death-ligand 1 (PD-L1) expression was affected in 67 patients (45.8%); it was <50% in 57 patients representing 85% of cases and ≥50% was noted in 10 patients (15%) (Table 2).

**Table 2:** Molecular profile EGFR: Epithelial Growth Factor Receptor; ALK: Anaplastic Lymphoma Kinase; ROS1: Reactive Oxygen Species; PD-L1 : Programmed Death-Ligand 1

	Cases (N=146)	Percentage %
Mutations		
EGFR		
Mutated	28	19.2
Not mutated	51	34.9
Unknown	67	45.9
ALK		
Mutated	6	4.1
Not mutated	59	40.4
Unknown	81	55.5
ROS		
Mutated	1	0.7
Not mutated	6	4.1
Unknown	139	95.2
PD-L1 expression		
PD-L1 ≥50%	10	6.8
PD-L1 <50%	57	39
Unknown	79	54.1

The time between histological confirmation and the start of treatment for the patients in our study was two months on average; it varied between 15 days and 12 months. Among the 146 patients, 16 were lost to follow-up (10.9%) and 11 female patients (7.5%) had received exclusive palliative supportive care (BSC: best supportive care). Four patients (stage IIB) were referred for surgery (2.7%), nine of the stage IIIB patients (6.1%) had received neoadjuvant chemotherapy followed by concomitant chemoradiotherapy (CCR), then placed under surveillance, and 106 stage IV patients (72.6%) had received first-line palliative systemic treatment (Table 3). Among these, 89 cases (84%) had received platinum-based chemotherapy (including 11 EGFR-mutated) and 17 EGFR-mutated (16%) had received anti-EGFR treatment.

**Table 3:** Protocols used in the first-line treatment

First-line protocols	Cases (N=106)	Percentage %
Carboplatin + Paclitaxel	79	74.52
Navelbine monotherapy	5	4.71
Carboplatin + Pemetrexed	2	1.88
Carboplatin + Gemcitabine	1	0.94
Cisplatin + Gemcitabine	1	0.94
Docetaxel monotherapy	1	0.94
Erlotinib	11	10.37
Gefitinib	6	5.66

Zoledronic acid was added to palliative systemic therapy in 11 patients (7.5% of cases) and bevacizumab in seven patients (4.7% of cases). The mean number of chemotherapy cycles received was 4 (1-10). For anti-EGFR therapy, the mean number of cycles was 7 (1-29). Among the 89 patients who received chemotherapy, six patients could not be assessed for response to treatment because they were lost to follow-up. Disease assessment was based on the response evaluation criteria in solid tumors (RECIST) criteria. Among the 83 patients with evaluable disease, 20 patients (24%) had a partial response, 31 patients (37%) had stability and 32 patients (39%) had progression. Regarding anti-EGFR treatment (n=17), three patients (18%) had a complete response, six patients had a partial response (35%), six patients had stability (35%) and two patients had progression (12%).

After progression, 32 patients received second-line systemic treatment. Twenty-three patients (72%) received chemotherapy and nine patients (28%) received anti-EGFR (Table 4).

**Table 4:** Protocols used in the second-line treatment

Second-line protocols	Cases (N=32)	Percentage %
Docetaxel monotherapy	10	31.25
Navelbine monotherapy	7	21.87
Pemetrexed monotherapy	5	15.62
Gemcitabine monotherapy	1	3.12
Erlotinib	9	28.12

Palliative radiotherapy was administered to 25 female patients, five received decompressive spinal radiotherapy, 10 received cerebral radiotherapy, one received consolidation and analgesic radiotherapy to the femoral neck, and nine received palliative pulmonary radiotherapy. The treatments were tailored according to tumor location, symptom severity, and overall patient condition, aiming to relieve symptoms and improve quality of life.

**Figure 1:**
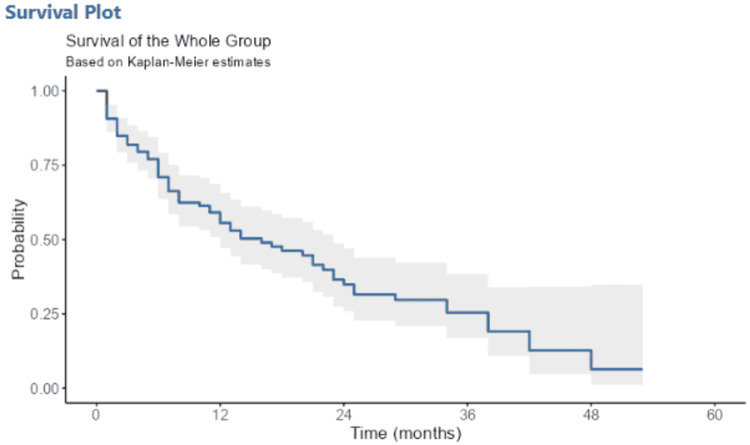
Time to death survival curve 12-month survival is 57% [48%-67%, 95% CI] 36-month survival is 26% [17%-39%, 95% CI]

**Figure 2:**
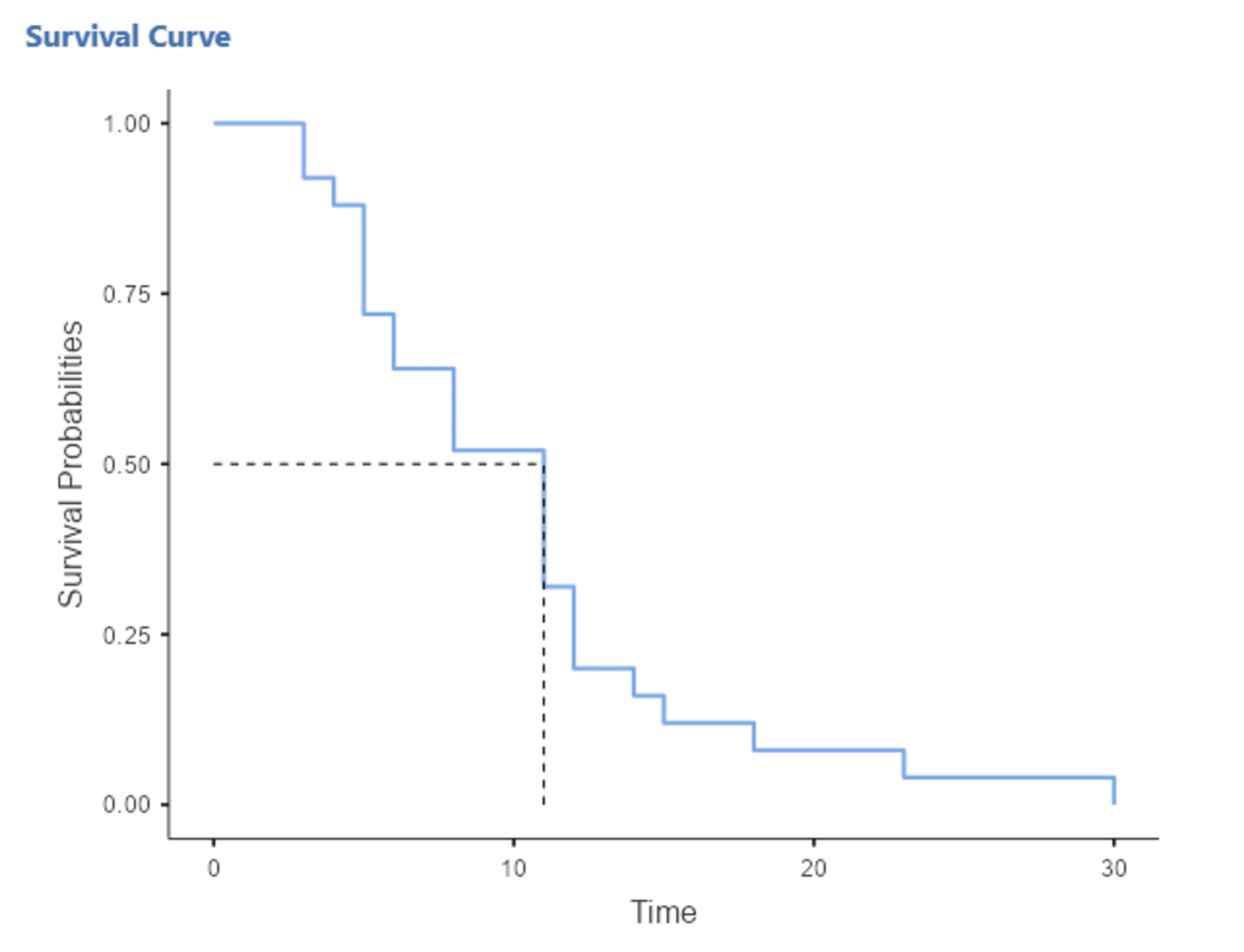
Time to progression survival curve

In our series, the median follow-up was seven months (1-53 months), the median overall survival (OS) was 17 months (12.23) (Figure 1) and the median progression-free survival (PFS) was 11 months (6.12) (Figure 2). Furthermore, the one-year and three-year OS rates were 57% and 26%, respectively (Figure 1).

**Figure 3:**
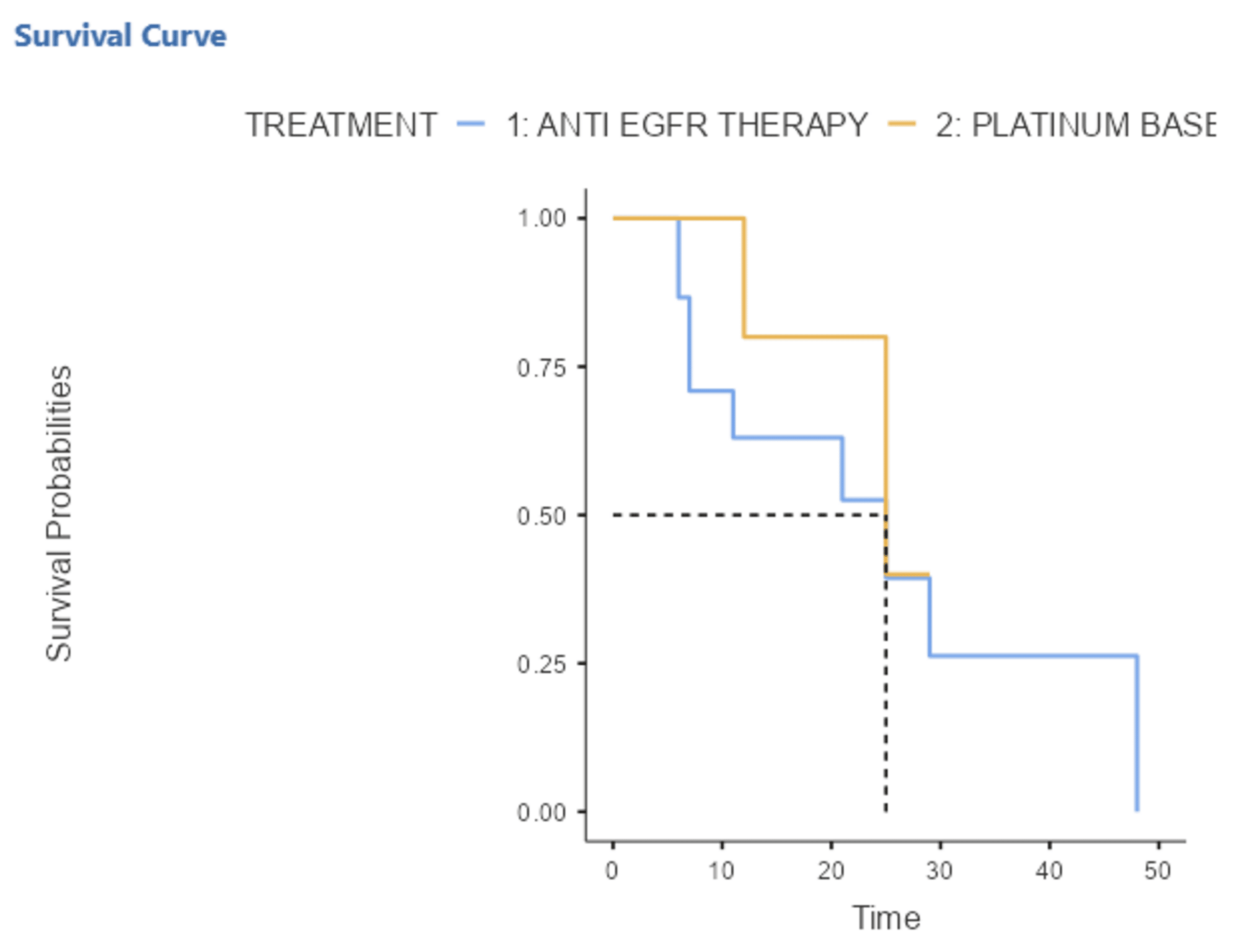
Kaplan–Meier curve representing the overall survival of EGFR-mutated patients treated with anti-EGFR and platinum-based chemotherapy 1: The overall survival (OS) in the group of EGFR-mutated patients treated with anti-EGFR therapy 2: The overall survival (OS) in the group of EGFR-mutated patients treated with platinum-based chemotherapy EGFR: Epithelial Growth Factor Receptor

The median OS in the group of EGFR-mutated patients treated with anti-EGFR was 25 months (11-47) and in the group of EGFR-mutated patients treated with platinum-based chemotherapy was also 25 months (3-25). This median OS in the anti-EGFR therapy group was similar to that in the platinum-based chemotherapy group with a statistically non-significant value of p = 0.32 (Figure 3).

**Figure 4:**
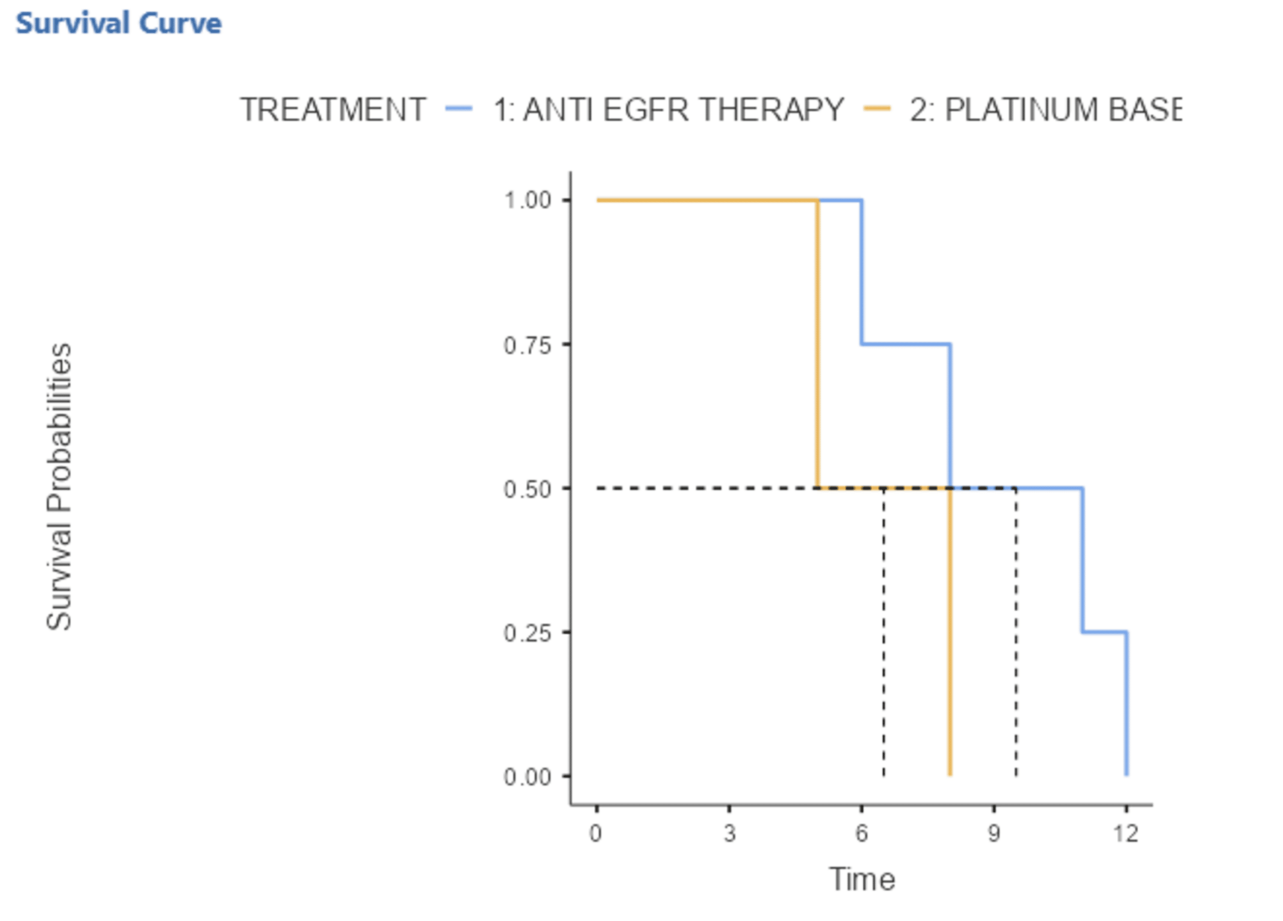
Kaplan–Meier curve representing progression-free survival of EGFR-mutated patients treated with anti-EGFR and platinum-based chemotherapy 1: The progression-free survival (PFS) of EGFR-mutated patients treated with anti-EGFR therapy 2: The progression-free survival (PFS) of EGFR-mutated patients treated with platinum-based chemotherapy EGFR: Epithelial Growth Factor Receptor

Similarly, the median PFS in the group of EGFR-mutated patients treated with anti-EGFR was 9.5 months (6-12) while in the group of EGFR-mutated patients treated with platinum-based chemotherapy, it was 6.5 months (5-8). The median PFS in the anti-EGFR therapy group was slightly longer, but statistically non-significant than that in the platinum chemotherapy group with a p = 0.22. It was also noted that the OS and PFS curves of chemotherapy treatment fell more rapidly compared to those of anti-EGFR treatment, meaning that patient death occurred very rapidly under chemotherapy (Figure 4). 

## Discussion

Lung cancer is one of the leading causes of cancer worldwide, accounting for 11.4% of cancer cases [13]. Women in developed countries had a higher mortality rate from lung cancer, with lung cancer deaths ranked second after breast cancer [14]. In our series, the mean age of patients was 63.5 years. This average is similar to that reported in Switzerland (62 years) [15], and slightly higher than that reported in Morocco in 2015 (58.3 years) [16], Indonesia (56.9 years) [17] and Italy (60 years) [18]. Furthermore, in our study, the age ranges were between 31 and 86 years, indicating that lung cancer affects both young and old women.

The majority of patients (87.7%) were non-smokers and 7.5% were exposed to passive smoking. These results are in line with those of Zakkouri et al. [19] who reported that smoking does not explain all cases of lung cancer in women, because 75% of patients have never smoked and 14% have been exposed to passive smoking. Passive smoking is a risk factor for lung cancer in women that is often mentioned in the literature. According to Sisti and Boffetta [20], passive smoking is implicated in women in 14.2% of cases. However, the relative risk differs according to the type of exposure: family, occupational, or social [21], and according to the intensity and duration of passive smoking [22].

5.5% of patients in our study were treated for pulmonary tuberculosis. Pulmonary infections increase the risk of lung cancer due to prolonged inflammation, pulmonary fibrosis and pulmonary tissue damage [23,24]. A history of lung cancer was present in 2.7% of cases in our study. The International Lung Cancer Consortium [25] showed that there is a 1.5 times greater risk of lung cancer in people with a first-degree relative (mother, father, brother or sister) suffering from lung cancer, which would highlight the hereditary nature of the disease. In European and American series, most cases of non-small cell lung cancer were observed at a relatively advanced stage according to the TNM classification system [26], as in the study of Zakkouri et al. [19]. In our population, the proportion of non-small cell lung cancer at stage IV was higher (87%), 11.6% of cases were at stage III, only 1.4% were at stage II and no patient was operated on at stage I. This high percentage of advanced stage can be explained by the delay in diagnosis.

Adenocarcinoma is the most predominant histological type in women with non-small cell lung cancer [27,28]. In our series, adenocarcinoma (90.4%) was also the most frequently diagnosed histological type in patients with NSCLC compared with squamous cell carcinoma. These results are similar to those reported in a study in the USA that showed that 71% of lung cancers in women were adenocarcinomas [29]. Similarly, a meta-analysis of 17 studies revealed that the incidence of adenocarcinoma was 3.4 times higher than squamous cell carcinoma in non-smoking patients and that up to 60% of lung cancers in women were classified as adenocarcinomas [30].

In our study, the frequency of EGFR mutation was high (35.4%). This frequency is close to that obtained in other studies in women with NSCLC nationally and in Turkey and Lebanon [19,31,32]. In Europe and America, this frequency is generally lower [18,33-36]. However, in Asian women with NSCLC, the frequency of EGFR mutation is very high [37,38]. Moreover, the most frequent EGFR mutation was in exon 19 (86%). This distribution is higher than that obtained in other series [18,39]. The most common mutations associated with sensitivity to EGFR tyrosine kinase inhibitors (TKIs) include the exon 19 mutation and the L858R point mutation in exon 21 [40].

ALK mutation was sought in 65 patients and was positive in six patients (9.2%), and ROS mutation was requested for seven patients, which was positive in one case (14.2%). The frequency of ALK mutation is little studied nationally. In a study conducted in our institution [41], this mutation was sought in 28 women and was negative in all of them. The use of ALK inhibitors in patients with NSCLC with ALK mutation has demonstrated an impressive response rate and progression-free survival compared to chemotherapy [42].

Furthermore, in our series PD-L1 expression was in 39% <50% and 6.8% ≥ 50%, four patients received immunotherapy (Pembrolizumab). The history of immunotherapy could be different. A 2018 meta-analysis [43] found that, compared with controls, men treated with immune checkpoint inhibitors (ICIs) had a significantly reduced risk of death and greater benefit than women. Regardless of the histological type of cancer, line of treatment, type of ICI given, and type of control treatment, men also had greater efficacy of ICIs, while another group [44] of researchers found that the combination of chemotherapy with ICIs had a greater beneficial effect on women with advanced cancer than on men.

For stage IV, first-line chemotherapy consists of a combination of platinum salts and a third-generation drug (gemcitabine, paclitaxel, docetaxel, irinotecan, vinorelbine) for PS 0-1 patients. For those with PS 2, monotherapy or non-platinum combination therapy is recommended, while patients with PS 3 require palliative care [45]. In our series, 106 stage IV patients (72.6%) had received first-line palliative systemic treatment. Among these, 89 cases (84%) had received platinum-based chemotherapy (including 11 EGFR mutated) and 17 EGFR mutated (16%) had received anti-EGFR therapy (Erlotinib or Gefitinib). Palliative radiotherapy aims to alleviate pain or relieve symptoms of the disease. In our series, 17.1% of patients benefited from this therapeutic modality, compared to 17.7% in the series of Debieuvre [46] and 22% in that of Radzikowska [47].

The median OS was 17 months (12.23) and the median PFS was 11 months (6.12); the overall survival rate at 12 months was 57%. This rate is higher than those obtained in studies conducted in our institution in 2016 (32.3%) [48] and in Brazil (34.5%) [49]. This improvement in survival can be explained by the generalization of the compulsory health insurance scheme in December 2022. To assess the differences in response to chemotherapy, Albain et al. [50] found that women treated with platinum salts survived longer, with a 14% reduced risk of death, a notable difference in women aged 60 and over. Similarly, Wakelee et al. [51] compared female and male patients in the Eastern Cooperative Oncology Group trial and found that the overall median survival was significantly longer in women (9.2 months versus 7.3 months).

In second-line treatment, monotherapy or TKIs are used [45]. In our series, 32 patients had received second-line systemic treatment. 71.8% had received chemotherapy (the majority of which are docetaxel, Navelbine, pemetrexed, gemcitabine) and 28.1%TKIs (Erlotinib). Several studies have shown that female gender is predictive of better response to EGFR TKIs (gefitinib or erlotinib), particularly in patients with NSCLC. The IDEAL 1 and IDEAL 2 studies demonstrated that female gender, adenocarcinoma, and non-smoking were predictors of better responses [52,53]. In the ENSURE study, which examined 217 patients, 61% of whom were women, with stage IIIB/IV EGFR-mutated NSCLC, comparing erlotinib to a cisplatin doublet, the median PFS was 11 months in the erlotinib group versus 5.5 months in the chemotherapy group [54].

The EURTAC trial [55] in Europe, in patients with EGFR-mutated NSCLC, 72.4% of whom were women, compared erlotinib to platinum-based chemotherapy in first-line metastatic treatment. The results revealed a PFS of 5.2 months in the platinum-based chemotherapy arm versus 9.7 months in the erlotinib arm. The response rate was 10.5% under chemotherapy versus 54.5% under erlotinib, and the median overall survival was 18.8 months for chemotherapy compared with 22.9 months for erlotinib.

In our study, the median PFS was slightly longer, statistically non-significant, in the group of EGFR-mutated patients who received anti-EGFR therapy (9.5 months) compared with the group of EGFR-mutated patients receiving platinum-based chemotherapy (6.5 months). This PFS is close to those obtained in Italy, China and Hungary, Turkey and Latvia, in which the median PFS of female patients treated with EGFR TKIs were 11.4, 12.4 and 13.2 months respectively [56-58]. The median PFS of our study can be explained by the limited number of EGFR-mutated patients. Similarly, the median OS was similar for EGFR-mutated patients treated with anti-EGFR therapy (erlotinib or gefitinib) compared with those of EGFR-mutated patients who received platinum-based chemotherapy (25 months). This median SG is lower than that obtained in Korea (30.3 months) [59] but much higher than those reported in Asia (12 months) [60] and in the Netherlands (12.4 months) [61].

This study has several limitations. Its retrospective design introduces potential selection bias and incomplete clinical data, which may affect the strength of the conclusions. The absence of a control group, particularly healthy controls, also limits the comparative value of the findings. In addition, the small number of patients with EGFR-mutated tumors who received anti-EGFR therapy reduces statistical power and restricts robust subgroup analyses. Finally, variability in clinical practice and the single-center nature of the study may limit the generalizability of the results.

## Conclusions

Lung cancer remains relatively rare in women, and in our cohort, most patients were never-smokers (87.7%), with adenocarcinoma as the predominant histological type (90.4%) and nearly 87% diagnosed at an advanced, non-surgical stage, underscoring the need for improved prevention and early detection strategies. Median PFS was slightly longer with EGFR TKIs compared to platinum-based chemotherapy, supporting systematic EGFR mutation testing to guide targeted therapy. Future research should aim to clarify female-specific risk factors, genetic, hormonal, and environmental, while developing more effective screening approaches and evaluating long-term outcomes and optimal therapeutic sequencing in women with EGFR-mutated tumors to advance personalized treatment and improve survival.
